# It never rains but it pours: COVID-19 pandemic impact on mental health in older adults

**DOI:** 10.1093/eurpub/ckac129.414

**Published:** 2022-10-25

**Authors:** A Amerio, C Stival, A Lugo, T Fanucchi, L Cavalieri d'Oro, L Iacoviello, A Odone, A Zucchi, S Gallus, G Serafini

**Affiliations:** 1 DINOGMI, University of Genoa, Genoa, Italy; 2 IRCCS San Martino Polyclinic Hospital, Genoa, Italy; 3 Department of Environmental Health Sciences, IRCCS Mario Negri Institute for Pharmacologic Research, Milan, Italy; 4 Unit of Alcohology, Careggi Hospital, Firenze, Italy; 5 Brianza Health Protection Agency, Monza, Italy; 6 EPIMED, Insubria University, Varese, Italy; 7 Department of Epidemiology and Prevention, IRCCS Neuromed, Pozzilli, Italy; 8 Department of Public Health, University of Pavia, Pavia, Italy; 9 School of Medicine, Vita-Salute San Raffaele University, Milan, Italy; 10 Bergamo Health Protection Agency, Bergamo, Italy

## Abstract

Italy was the first country to be hit by the 2019 coronavirus disease (COVID-19) in Europe holding one of the highest clinical burdens. Older adults are those paying the highest price for the COVID-19 emergency. Within the Lost in Lombardy project, a web-based cross-sectional study assessing the prevalence of depressive and anxiety symptoms, hopelessness and insomnia before and during the COVID-19 pandemic, was conducted on a representative sample of 4,400 older adults aged 65 years or more from the Lombardy region recruited between November 17th and 30th 2020. The prevalence of depressive symptoms increased by + 112% during the pandemic, anxiety symptoms by + 136%, insufficient sleep by + 12%, unsatisfactory sleep by + 15%. Feelings of hopelessness were more frequent among women compared to men (15.1% vs. 10.4%) and increased with increasing age. A worsening in each of the four specific mental health outcomes was more frequently observed in women (OR = 1.50, depression; OR = 1.31, anxiety; OR = 1.57, sleep quality; OR = 1.38, sleep quantity), in subjects who decreased their physical activity during the pandemic (OR = 1.64, depression; OR = 1.48, anxiety; OR = 2.05, sleep quality; OR = 1.28, sleep quantity), and with increasing number of pre-existing chronic diseases (p for trend<0.001 for depression and anxiety; p for trend=0.010 for sleep quality; p for trend=0.012 for sleep quantity). A worsening in depressive symptoms was more frequently observed in more educated subjects (p for trend=0.008), while a worsening in anxiety symptoms in subjects living in main towns compared to outskirt with an economic status below the mean. The use of at least one psychotropic drug - mostly antidepressants/anxiolytics - increased by + 26% compared to pre-pandemic. The protection of the mental health status of this vulnerable segment of population needs to be recognized as a real public health priority.

